# Commentary: Invited review: glucosinolates might result in low methane emissions from ruminants fed brassica forages

**DOI:** 10.3389/fvets.2023.1227500

**Published:** 2023-10-06

**Authors:** Luca Todini, Francesco Fantuz

**Affiliations:** School of Biosciences and Veterinary Medicine, University of Camerino, Matelica, Italy

**Keywords:** Digesta Retention Time, free triiodothyronine, greenhouse gas, physiological parameters, plant secondary compounds, rumen

## Introduction

The present commentary aims to clarify some main physiological concepts and literature findings that may be subject to confusion arising from the invited review (1) (referred to as “TIR” hereafter), published in 2020 in this journal *Frontiers in Veterinary Science*.

The purpose of TIR was “to summarize literature reports on the use of brassica forage to mitigate CH_4_ emissions … and highlight the possible role of glucosinolates”. The articles quoted in TIR indicate that “ruminants fed forage brassica emit low methane”, and that “a shorter mean retention time of particulate and liquid digesta is associated with less CH_4_ yield”.

Then, the “Effects of Secondary Metabolites in Brassica Forages on Triiodothyronine” and “The effects of Free Triiodothyronine on Digesta Retention Time and on Methane Emissions” are reviewed.

## Literature cited

Several concerns arise when TIR faces “Effects of Secondary Metabolites in Brassica Forages on Triiodothyronine”. The topic of Brassicas, glucosinolates (GLS), and thyroid function is challenging, and the related literature is really extensive. The present Commentary considers articles cited by TIR, and we believe that certain results from these articles have not been adequately considered, leading to an unsupported hypothesis.

First, TIR states that “S-methyl-L-cysteine sulfoxide can increase ghrelin and thyroid hormones in the plasma (12)”. However, in that cited review (2), it is stated that “kale feeding is associated with increased blood plasma concentrations of growth hormone and thyroxine” (not ghrelin and thyroid hormones). This sentence refers to previous experiments by the same author [(3, 4); the latter is not cited by TIR], comparing diets (kale vs. ryegrass-clover pasture) and iodine supplementation (with vs. without). In the presence of supplementary iodine (“to counteract the goitrogenic properties of the inorganic thiocyanate ion released on hydrolysis of GLS”), kale feeding was associated with an elevation in the plasma concentration of T_4_. When iodine supplementation compensated for the anti-thyroidal actions of GLS, S-methyl-L-cysteine sulfoxide (SMCO) was hypothesized to have a stimulatory effect on T_4_ increase. However, to the best of our knowledge, this hypothesis was not further investigated. It should be noted that T_3_ (triiodothyronine) remained unaffected by the diet. As stated in TIR, “it is unlikely that SMCO has an effect on blood FT_3_ concentration”. When growing lambs were fed kale without iodine supplementation, concentrations of both thyroid hormones (TH) dropped (less markedly for T_3_), as expected and reported (4).

Comparably, serum T_4_ concentrations decreased in lambs grazing Brassicas, which were restored by iodine supplementation (5). Again, serum T_3_ levels remained unaffected by the diets (5). Unfortunately, the citation of this latter article in TIR appears to be non-compliant, as it states that “For example, feeding turnip (*Brassica rapa* L.) and kale (*B. oleracea* L. var. *acephala* DC) to fattening lambs can increase the concentrations of T_3_ and T_4_ in serum (76)”.

Similar findings were observed in calves fed rapeseed–mustard cakes with high or low GLS contents (6): “T_3_ remained within the normal range”, while T_4_ decreased in a dose-dependent manner. Unfortunately, these results, reported in the original article as “thyroxine changed quadratically with increasing glucosinolate levels”, were reported in TIR as “quadratically increased serum T_4_ concentration”.

Goitrogenic effects of GSL, and therefore their ability to decrease T_4_ secretion by the gland and TH blood concentrations, are widely confirmed by the literature cited in TIR, involving pigs (7), mares (8), turkeys (9), and sheep (10, 11).

Among the references mentioned in TIR, only the abstract is available for (12), stating that “The sheep receiving GLS and nitrates without iodine and selenium supplementation developed enlargement of the thyroid gland, … low T_4_ level…, high T_3_ level and a narrow T_3_/T_4_ ratio”. The full text of (13), dealing with rats, is not available.

## Discussion

The thyroid gland primarily secretes T_4_, making blood T_4_ concentrations indicative of the synthesizing and secretory rates of the gland. On the other hand, T_3_ secretion by the thyroid gland is minimal, and the majority of circulating T_3_ is derived from the activation of T_4_ to T_3_ by deiodinase enzymes at the peripheral level. Therefore, circulating T_3_ concentrations reflect the rate of deiodinase activity, representing the balance between peripheral enzymes involved in activating and inactivating T_4_.

The data from articles cited by TIR are consistent with basic physiological knowledge regarding the effects of GLS and iodine on thyroid gland activity and TH blood concentrations. It has been well-established for decades that GLS derivatives inhibit TH synthesis and secretion, interfering with various steps of the process in a dose-dependent manner. The main effects involve the inhibition of the thyroperoxidase (TPO) enzyme, leading to the impairment of iodide uptake, oxidation, binding to thyroglobulin, and hormone secretion. Moreover, such goitrogenic compounds interfere with the deiodination of T_4_ in peripheral tissues. Many literature articles on various species have reported that iodine supplementation can at least partially restore physiological functions and parameters, including circulating TH, impaired by GLS intake.

Among mammals, ruminants are less sensitive to these effects than monogastric species due to greater degradation of the active compounds in the forestomach environment. Regarding the effects on blood TH concentrations and T3/T4 ratio, it is important to consider the quality and amounts of Brassica forages included in the diets (and iodine intake, as well). Recently, in dairy cows, no effect was found on T3 replacing 25% of DM with Brassicas (14), nor on T3 and T4 with 30 and 45% forage rape (15).

In conclusion, literature data are often contradictory and do not seem to support the hypotheses proposed in TIR (“GSLs and/or their breakdown products in brassica forage crops … increase blood FT_3_ concentration in ruminants”). Furthermore, the “Concluding remarks” (“GSL and its metabolites can elevate the concentration of FT_3_ in ruminants”) and the “Abstract” (“It is reported that feeding brassica forages to sheep can increase the concentration of free triiodothyronine (FT_3_) in serum. We hypothesize that GSLs or their breakdown products may stimulate the secretion of thyroid hormone FT_3_ in ruminants”) is not substantiated by the cited references. Finally, in Figure 4, the first step of the diagram appears to lack support from the references cited.

## Author contributions

All authors listed have made a substantial, direct, and intellectual contribution to the work and approved it for publication.
